# How automated machines influence employment in manufacturing enterprises?

**DOI:** 10.1371/journal.pone.0299194

**Published:** 2024-03-05

**Authors:** Hong Jiang, Yingfan Ge, Chunhao Yang, Hongxin Yu

**Affiliations:** 1 School of Management, Shanghai University of Engineering Science, Shanghai, China; 2 School of Finance, Shanghai University of Finance and Economics, Shanghai, China; 3 Faculty of Business Economics, Shanghai Business School, Shanghai, China; King Khalid University, SAUDI ARABIA

## Abstract

This paper theoretically analyzes and empirically examines the impact and mechanisms of automated machines on employment in manufacturing enterprises, drawing on task-based model and using micro data from listed Chinese manufacturing enterprises between 2012 and 2019. Our findings reveal that: (1) Automated machines in manufacturing enterprises leads to a substitution effect on the total labor force, with a substitution effect on low-skilled labor and a creation effect on high-skilled labor in terms of employment structure. (2) Further analysis indicates that automated machines primarily have a positive effect on R&D and technical staff, a non-significant effect on sales staff, and a negative impact on production, administrative, and financial staff. (3) The primary influencing mechanisms of automated machines on employment in manufacturing firms are productivity effects and output scale effects, based on the mediation effect model. (4) Considering the industry linkage effect, we employ the input-output method and the Input-Output Table and find that automated machines for upstream (downstream) manufacturing enterprises will result in a substitution effect on employment for downstream (upstream) enterprises. The novelties and research contributions are as follows: (1) we conduct a structural decomposition of total employment, and further decompose employment positions into production, R&D, sales, finance, and administration. (2) We try to investigate the industry linkage effect about the impact of automated machines on the employment of upstream and downstream enterprises. (3) We use data from listed manufacturing companies, and the data of existing research are about provincial and industry-level data.

## 1. Introduction

With the exacerbation of the aging problem, rising labor costs, the trend of "re-industrialization", and the global boom in artificial intelligence technology, the input of automated machines is increasing worldwide, especially in China, where the scale of industrial robots increased significantly from 2010 to 2019. Data from the International Federation of Robots (IFR) shows that China has been the world leader since 2016, with the scale of industrial robots reaching 2,834,500 units until 2019, surpassing figures from the US, Japan, and Germany. The global outbreak and normalization of Covid-19, along with the strong support of governments for automation technologies such as artificial intelligence in recent years, have further accelerated the input and technological progress of automated machines such as artificial intelligence. However, at the same time, concerns have arisen regarding the impact of AI and other automation technologies on the labor market. Many literatures suggested that AI and other automation technologies can significantly reduce labor demand [1, 2]. Research on the impact of technological progress on the labor market has a long history. As early as the first half of the 20th century, many scholars believed that technological progress would lead to a decline in labor demand [3, 4]. However, historical evidence from past industrialization shows that while machines did cause small-scale short-term unemployment and structural adjustments in the labor market, but they did not lead to widespread long-term unemployment [5]. Therefore, it is crucial to carefully examine whether the automated machines will have an impact on the labor market, including its effect on labor demand and structure, as well as the underlying mechanisms, and there is an urgent need for sufficient evidence from theoretical and empirical studies. Additionally, understanding the impact on labor employment in both upstream and downstream enterprises is essential. This is crucial for gaining a comprehensive understanding of the current state of automation machines in China and for the development of appropriate industrial and employment policies. This paper aims to study whether the automated machines will have an impact on the employment in manufacturing and the influencing mechanisms.

The impact of automated machines on labor force employment remains a hotly debated topic without conclusive findings. Some scholars argue that automated machines lead to a substitution effect on certain jobs [1]. Additionally, several studies suggest that automation has a creation effect on high-level and complex labor, and a disruptive effect on low-skilled labor [1, 2, 6, 7]. Furthermore, some scholars also believe that with the rapid development of automation technology and digital technology, the trend of "de-skilling" becomes more prominent, leading to an increased substitution effect of automated machines on high-skilled labor [8]. To investigate the impact mechanism, existing studies are mainly based on a task-based model for theoretical research. These models suggest that the automated machines affect the productivity of different types of labor and have an impact on the demand for different jobs, consequently affecting employment in different job positions as well as overall employment levels [1, 6]. Therefore, there have been more theoretical derivations than empirical tests on microdata, focusing on the impact of automated machines on total employment rate in an economy or employment in a specific industry (such as manufacturing), and less emphasis on the impact of automated machines on employment in upstream and downstream industries from the perspective of industry linkages. Additionally, existing literature classifies jobs into high-skilled and low-skilled positions, further decomposition is necessary to account for the latest development characteristics of automation technology. For example, although sales positions that focus on customer communication and service are not high-skilled labor, the impact of automated machines on them is not necessarily a substitution effect.

This paper examines the impact of the automated machine on employment in the manufacturing sector and explores the underlying mechanisms using microdata from listed manufacturing firms from 2012 to 2019, drawing on a task-based model. Based on Input-Output Tables, this paper quantifies the level of automated machines in upstream and downstream manufacturing enterprises. Furthermore, this study examines the impact of automated machines in upstream (downstream) manufacturing enterprises on the labor demand of downstream (upstream) enterprises. The empirical results show that the automated machines in manufacturing industry have a significant impact on labor force employment in enterprises, mainly in the form of the destruction effect on low-skilled labor force and the creation effect on high-skilled labor force. The substitution effect of automated machines in manufacturing firms is driven by two primary mechanisms: the expansion effect on output and the enhancement effect on productivity. Moreover, the improvement of the technological level of automated machines in upstream (downstream) manufacturing enterprises will intensify the substitution effect on employment for downstream (upstream) enterprises.

This paper strives to introduce innovation in the following aspects. (1) Innovation in research content: Most of the existing literature focuses on the macro-level impact of automation technology on the labor market, lacking in-depth analysis of its impact mechanism and lacking research on the impact of automated machines on employment structure. This paper provides a comprehensive analysis of the channels through which automated machines in manufacturing industries affect employment. Additionally, it further decomposes employment positions into production, R&D, sales, finance, and management, aiming to study the impact of automated machines on employment in different positions. (2) Innovation in research perspective: Few studies have examined the impact of automated machines on the labor market of upstream (downstream) industries from an industry-linked perspective. Based on the Input-Output Table, this paper investigates the impact of automated machines on the employment of upstream and downstream enterprises. (3) Innovation in empirical data: Existing research on the impact of automated machines on the labor market often relies on provincial and industry-level data, lacking evidence based on microdata from individual enterprises. In contrast, we use data from listed manufacturing companies to conduct empirical testing, allowing for a more nuanced understanding of the heterogeneous characteristics of different enterprises.

The remaining structure of this paper is organized as follows: the second part provides a literature review and hypothesis, the third part outlines the research design, the fourth part presents the empirical results, the fifth part offers a discussion, and the sixth part is conclusion and implications.

## 2. Literature review and hypotheses

Since current automated machines mainly carry automated and intelligent technologies, this paper analyzes the impact of automated machines on employment by combining the literature about the impact of artificial intelligence, industrial robotics, and automation technology on the labor market.

Numerous studies have shown that automated machines have a substitution effect on total employment. Concerns about unemployment due to automation can be traced back to the early 19th century, when British textile workers destroyed machinery out of fear of being replaced by it. The history of industrialization shows that automation has led to a surge in unemployment rates in the short term [9, 10]. With the innovation of automation technology and the rapid development of artificial intelligence technology, more technologically advanced, intelligent automated machines will accelerate the impact on a larger scale of the workforce in more fields [11, 12]. Frey and Osborne (2015) argue that intelligent robots will replace 77% of jobs in China, 69% in India, 85% in Ethiopia, 55% in Uzbekistan, and 47% in the United States [8]. Susskind (2017) found, based on the task model, that the use of intelligent machines reduces relative wages and the share of labor income, which leads to high unemployment [12]. Acemoglu and Restrepo (2017), based on IFR data, found that for every additional robot per thousand workers in the United States, the employment-to-population ratio would fall by 0.18% to 0.34% and wages would reduce by 0.25% to 0.5%. Low-level, routine, repetitive, and programmed tasks are more likely to be automated, such as material handling, transportation, welding, and laser processing, and these tasks are primarily performed by low-skilled labor [13]. Therefore, automation is both more capable and more likely to replace low-skilled labor [14, 15]. The World Bank estimates that 57% of jobs in OECD countries are likely to be replaced by machines in the next 20 years, and developing countries are likely to be affected both by their automation technologies for labor substitution and by the retraction of manufacturing outsourcing in developed countries, and the risk of future employment deterioration will be much higher than in developed countries, especially in the manufacturing sector [16, 17]. For many developing countries, labor-intensive manufacturing plays a crucial role in economic development and employment security. However, the abundant low-skilled labor in labor-intensive manufacturing is more prone to being replaced by automated machines. Developing countries are more concerned about the substitution effect of automation on employment [18]. Some scholars have combined data from China and found a significant negative effect of robots on employment [19]. The automated machines have cost and efficiency advantages over labor in more positions, thus generating a substitution effect of labor in these positions. And the automated machines require a large amount of capital which brings out a significant cost effect, which leads to a decrease in the output scale in the short term, thus reducing labor demand [1, 6, 20].

However, many scholars believe that the compensatory effect of automated machines on employment can mitigate the substitution effect. The history of industrialization shows that although automated machines replace a large number of labors in the short run, they create a large number of new and high-level jobs in the long run [10, 21]. For example, while the introduction of personal computers (PCS) displaced jobs, new ones were also created. Some scholars have shown that since 1980, PCS have created 15.8 million jobs in the United States [10]. At the same time, the innovation of automated machines will create more new jobs. According to the World Economic Forum (2018), 100 million new jobs will be created between 2018 and 2022, adapting to the human-machine collaboration trend and complementing automation technologies. Bloom et al. estimate that, due to the extensive integration of artificial intelligence technology into daily life, between 2010 and 2030, the world will develop 734 million new jobs [22]. Acemoglu and Restrepo (2019), based on the task-based model, suggest that automation technology will reduce employment, and at the same time derive new employment opportunities by creating new job tasks (e.g., algorithm developers, machine trainers, smart device maintenance personnel, data annotators, etc.), and also will save the labor cost of enterprises, increase productivity, and expand the production scale of this industry and other industries based on spillover effects, which in turn will have a compensating effect on total employment [6]. Therefore, the impact of automated machines on employment is a combination of substitution and compensation effects [2, 7, 23]. The substitution effect between industrial robots and labor is more of a complementary substitution effect; labor supply shortage will force industrial firms to promote technological innovation, prompting them to apply more intelligent production to compensate for the negative impact of labor supply shortage. Many scholars, based on the capital-skills complementarity hypothesis, believe that automated machines are complementary to skilled labor. With the innovation of automation technology and the improvement of intelligence, there will be a greater demand for highly skilled labor that can complement automated machines, such as high-skilled labor in the fields of automation and intelligent research and design, equipment manufacturing and equipment application [24–26]. For instance, at the application level, every ten large intelligent robots require one artificial intelligence engineer, and smaller robots require even more engineers. Currently, nearly 1.5 million intelligent robots are in use globally, which equates to approximately 150,000 artificial intelligence engineers needed [5].

With the wide application of deep cognitive learning, image recognition, haptic perception, and other technologies in the automation field, the flexibility, precision, and intelligence of intelligent robots have been greatly improved, and automated machines can replace more complex positions and complete tasks beyond the scope of human physical and cognitive strength [5, 19], so the substitution effect on jobs is greater. Moreover, the change of automation technology is extensive and rapid, the progress of system and management is relatively slow, and education is lagging behind. Therefore, with the deepening of the progress of automation technology, the substitution effect on low-skilled labor with lower education is greater [5]. In Chinese manufacturing industry, the manufacturing workforce is more easily replaced by machines because of the large proportion of low-skilled labor and the low complexity and repeatability of the tasks performed by this workforce [19]. In addition, since upstream and downstream of manufacturing industries have a correlation effect, automated machines in upstream (downstream) will drive such technological progress in downstream (upstream) through chain transmission as well as technology spillover effects, which in turn will also have a substitution effect on labor in downstream (upstream). Hypotheses 1 and 2 are proposed in this paper:

Hypothesis H_1_: Automated machines in manufacturing will have a substitution effect on total employment, mainly in the form of destruction of low-skilled labor, however, the substitution effect can be mitigated by the creation effect on high-skilled labor.
Hypothesis H_2_: Upstream (downstream) automated machines will have a substitution effect on downstream (upstream) labor demand based on technology spillovers.

Studies on the influencing mechanisms of automated machines on employment have been conducted to show that automated machines replace low-level job tasks and generate emerging or high-level complex tasks, causing changes in firm productivity and output size, which in turn affect employment [1, 6]. (1) Productivity effect. The automated machines based on automated and intelligent machines and equipment and electronic equipment can replace low-level work tasks, and these automated machines perform tasks without time constraints, strictly programmed, and with higher quality and efficiency to complete the tasks compared to the labor force performing this type of tasks, so automated machines are more efficient advantage and will increase the productivity of the company [6]. In the meantime, automated machines will also create high-level job tasks that, by matching with a highly skilled workforce, will improve the productivity of automated machines [20]. However, increased enterprise productivity means a corresponding increase in the productivity of the workforce, with companies reducing the scale of employment in order to save labor costs; and as automation technologies become smarter, they have productivity advantages that can replace not only simple, repetitive manual labor positions (such as handling, transferring, welding, and laser processing), but also perform moderately complex and repetitive mental tasks (such as automated translation, driver lessness, intelligent factory management, voice, and image recognition, etc.), and in the future, even complex, creative mental labor jobs can be impacted. (2) Output scale effect. Since automated machines will replace low-level tasks as well as create new, high-level tasks, the initial cost of investing in automated machines will surge, and enterprises will need to upgrade labor skills as well as recruit and train highly skilled labor to perform high-level tasks and increase input in human capital to match automated machines, which will cause the cost pressure for enterprises in the short term and inhibit the expansion of their output scale. This leads to a crowding-out effect on enterprise labor demand. However, when input of automated machines increases to a certain level, it enhances firms’ profitability and stimulates them to expand their output scale, thus increasing the demand for labor [27, 28]. Moreover, with the development of automated machines, by replacing low-level work tasks, it is beneficial for enterprises to save production and operation costs, bringing about an output scale expansion effect and causing an increase in employment of enterprises. At the same time, this cost-saving effect also reduces the product price of enterprises, which increases the real income of consumers and stimulates consumer demand, resulting in the expansion of the output scale of enterprises and further increase of jobs [29]. This paper proposes hypothesis 3:

Hypothesis H_3_: Productivity effects and output scale effects are the main mechanisms of the impact of automated machines on employment.

## 3. Materials and methods

### 3.1 Sample selection and data sources

According to the availability and the quality of the data, we select the listed enterprises data in China’s manufacturing industry from 2012 to 2019. Since the topic of this paper is automation, the current literature mainly relies on the data of industrial robot ownership and new installation from the International Federation of Robotics (IFR) [13], the number of authorized patents related to automation technology at the industry level [30], and the share of working hours of workers using computer technology in each industry [2]. However, these data sets have limitations. The IFR industrial robot data set focuses on industrial robots, and the share of working hours of workers using computer technology mainly focuses on computer technology. This paper aims to explore the automation technology carried by automated machines, which covers a broader range of topics. Therefore, using these two data sets may be limited. Additionally, all three types of data used in current literature are at the industry level, which has a large data granularity. They cannot effectively analyze the impact of automated machines on micro-enterprise employment, nor can they facilitate research on the impact of automated machines on the employment of different types of labor in enterprises. Therefore, this paper selects data on automated machines from listed companies in the Choice database. Due to the serious lack of data on automated machines before 2012 and after 2019, data from 2012 to 2019 is selected.

And the data are obtained from Guotaian and Choice Oriental Fortune database. Data on the manufacturing industry is obtained from the official website of the National Bureau of Statistics of China and the China Statistical Yearbook. And the raw data are processed as follows: The data with obviously wrong key indicators were excluded, the sample of ST and ST* enterprises were excluded, the 1% and 99% quartiles of all variables were winsorized, and the total assets, R&D expenditures, average wages of employees, and revenues from enterprises’ foreign operations were deflated by price index.

### 3.2 Analytical techniques

To solve the problem of missing variables triggered by the change of time and individuals, we used a dual fixed effects model that controlled for firms and years in the baseline regression. As for mechanism testing, we used the widely adopted mediation effect model referred to Baron and Kenny (1986) [31]. To test the linkage effect of industry, we apply the idea of Input-output Method. To solve the problem of endogeneity, we controlled the interaction terms of time, year, and industry, which dealt with the problem of missing variables. And we construct an arithmetic average of technological level of automated machines at the industry level. We also adopted Bartik instrument variables to address the bidirectional causality problem referred to by Goldsmith-Pinkham et al. (2020) [32] and Zhao et al. (2021) [33]. For robustness, we used the placebo test method. And a two-stage least squares regression using *autom* lagged by one period (*L*. *autom*) as the instrumental variable [34]. We used STATA software to estimate all models.

### 3.3 Regression model and variable definitions

Based on the research hypothesis of this paper, the following regression model is developed in order to investigate the impact of automated machines in manufacturing on employment:

$$\text{ln}l_{it}=\alpha +\beta autom_{it}+\theta X+v_{i}+\varphi_{t}+\varepsilon_{it}$$ (1)

(1) Explained variables. *lnl*_*it*_ is the explanatory variable and refers to the size of employment in the firm, measured by using the number of employees in the firm. To further test the influence of automated machines on employment structure of manufacturing enterprises, we decompose the total employment into high- and low-skilled labor employment, which is represented by *Z*_*it*_, *L*_*it*_ respectively, where the high-skilled labor represents the R&D and technical workforces, and the other workforces are performed as low-skilled labor.
(2) Core explanatory variables. *autom*_*it*_ is the core explanatory variable, i.e., the technological level of automated machine in manufacturing enterprises, measured by the index of automated machine biased technical change based on the availability of data, referred to Jiang et al. (2022) [35].
(3) Control variables. *X* is a series of control variables, and control variables include the following factors: 1) The logarithmic value of the firm’s total assets (ln*asset*_*it*_), which is used to control the impact of productive resources owned by the firm on employment; the more productive resources, the more likely it increases the demand for labor. 2) The logarithmic value of firms’ R&D expenditures (ln*rd*_*it*_), which is used to control for the effect of firms’ innovation inputs on employment. 3) The logarithmic value of enterprise export size (ln*ex*_*it*_), which is used to control for the impact of enterprise external demand on employment. An increase in enterprise external demand will expand the production scale, which in turn will increase the demand for labor [23, 36]. This indicator is measured by revenues in Hong Kong, Macao, and Taiwan of China and abroad of enterprises because the data are disclosed in the annual reports of listed companies. 4) The logarithmic value of the average wage of employees of enterprises (ln*w*_*it*_), the average wage of employees is measured by the per capita salary of employees of listed companies, which is used to control the impact of labor cost on total employment in the manufacturing industry. The higher labor cost of enterprises, based on the cost-saving motive, will lead to a reduction in the demand for labor. In this paper, F-test and Hausman test were conducted on the regression model, and the test results rejected the mixed model regression and random effects estimation, and then the joint significance test of annual dummy variables was conducted, the P-value of the F-test is equal to 0, and the original hypothesis of "no time effect" is rejected, so this model controls for individual and time-fixed effects, set as *v*_*i*_, *φ*_*t*_, respectively.

## 4. Results

### 4.1 Descriptive analysis

The specific variables are described in Table 1. The descriptive statistics of the variables are shown in Table 2. The mean values of ln*L* and ln*Z* are 7.45 and 5.72 respectively, implying that the mean value of the number of low-skilled labor in manufacturing enterprises is much larger than that of high-skilled labor, and there is still more room for skill upgrading; The mean value of *autom* is 0.16 (bigger than 0), which means that the technological level of automated machines is improving. The mean value of ln*l* is 7.51, with a large difference between the minimum and maximum values and a standard deviation of 1.17, implying that the firm-level sample more accurately reflects the characteristics of automated machine.

**Table 1:** Description of relevant variables.

Variable Types	Variable Symbols	Meaning of variables
Explained variables	ln*l*_*it*_	Logarithmic value of employment
Core explanatory variables	*autom*_*it*_	The technological level of automated machine
Control variables	ln*asset*_*it*_	Logarithmic value of total assets
	ln*rd*_*it*_	Logarithmic value of R&D expenditures
	ln*ex*_*it*_	Logarithmic value of export size
	ln*w*_*it*_	Logarithmic value of average salary

**Table 2:** Descriptive statistics of variables.

Variable	Mean	SD	Min	Max
ln*l*	7.5125	1.1735	5.1180	10.6705
*autom*	0.1698	0.1935	0.0547	1.5574
ln*L*	7.4531	1.1820	4.9488	10.5725
ln*Z*	5.7247	1.1927	3.0910	8.9882
ln*asset*	21.4999	1.3550	15.3539	27.3493
ln*rd*	13.9736	1.7590	1.0276	22.4736
ln*ex*	18.9047	2.2391	6.0768	25.4970
ln*w*	11.4639	0.4470	5.0024	14.6230

### 4.2 Hypothesis testing

#### 4.2.1 Results of principal regression and analysis

**Fig 1:**
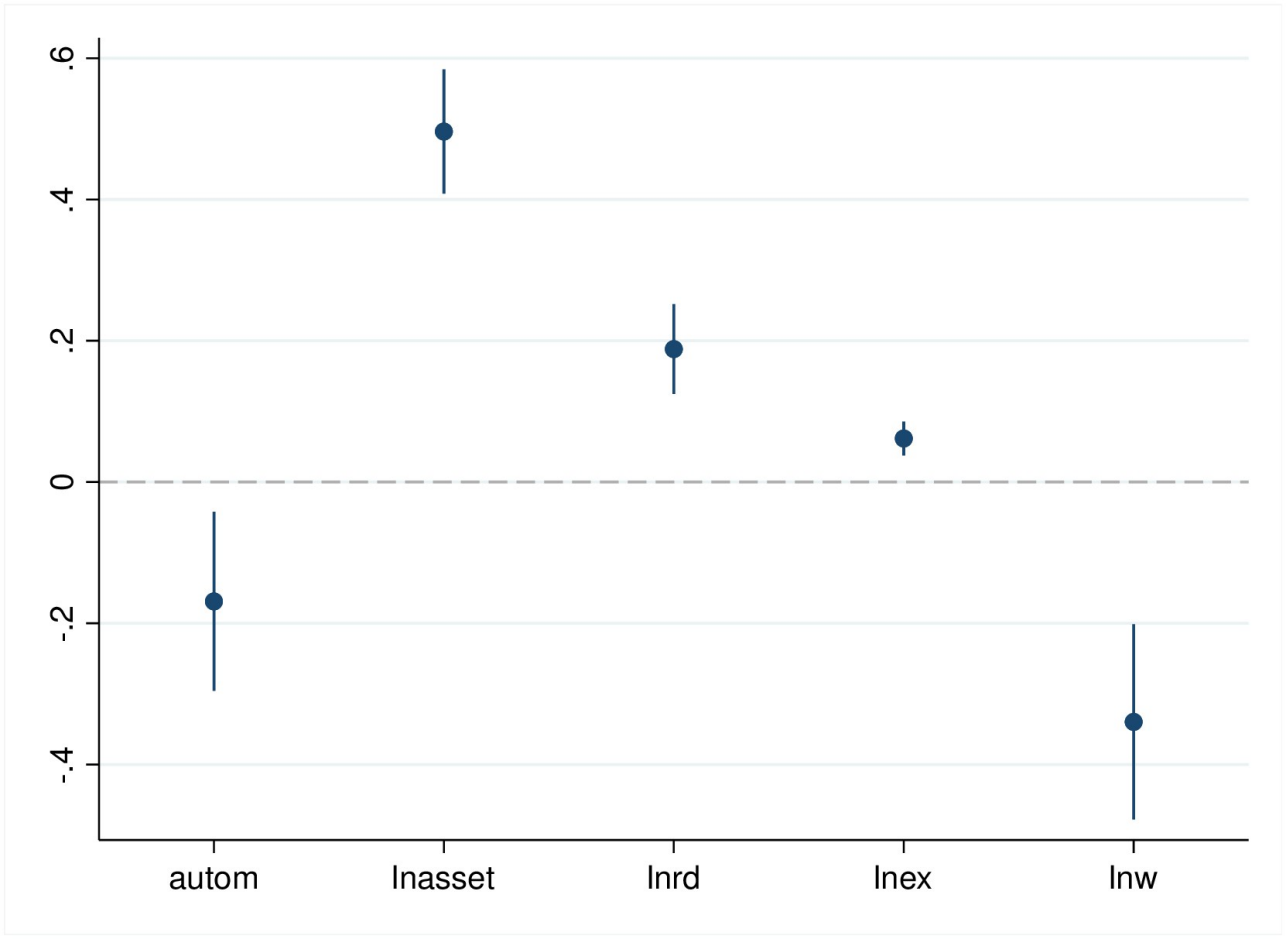
Estimated coefficients and confidence intervals of column 1 in Table 3.

**Fig 2:**
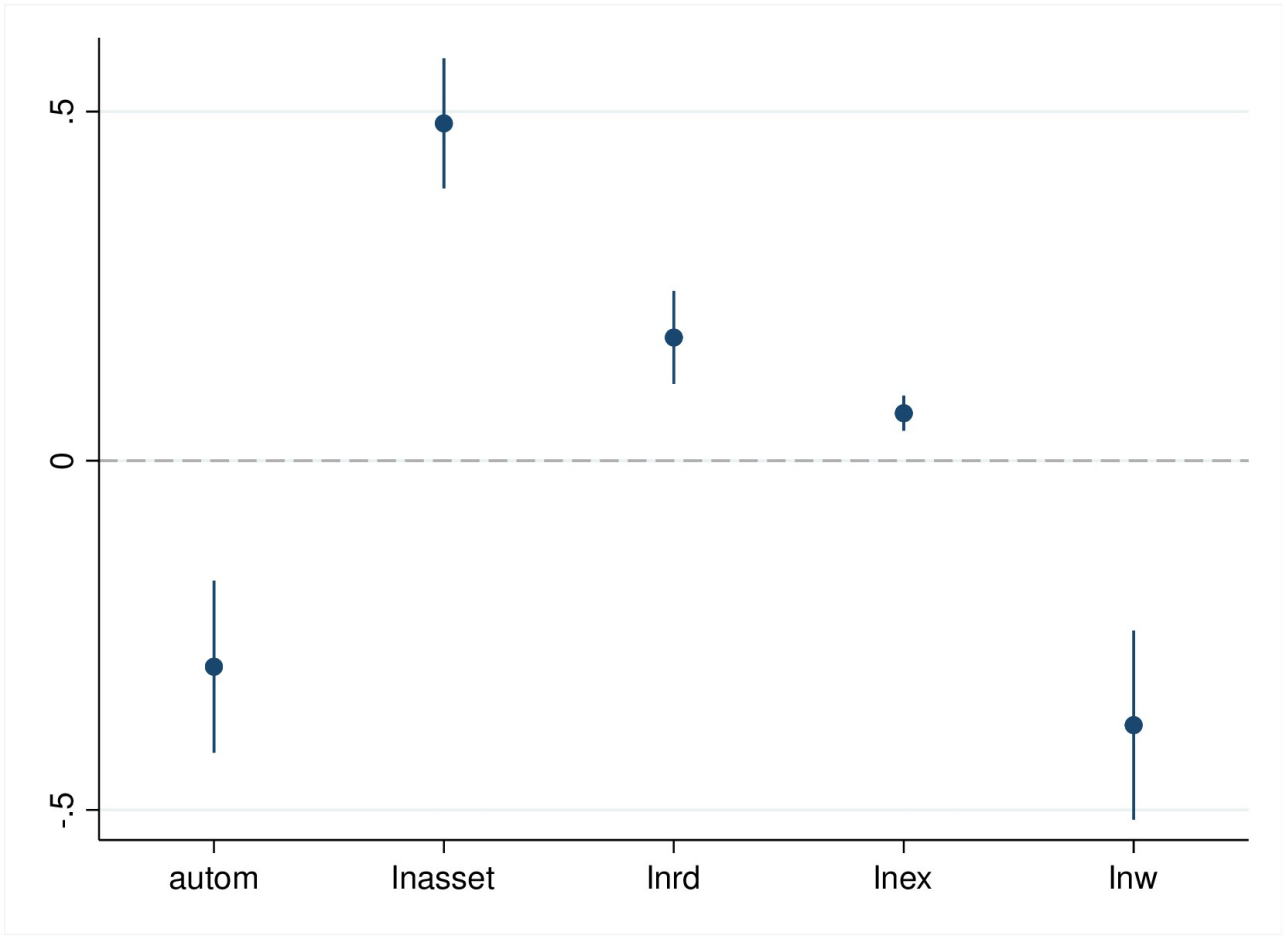
Estimated coefficients and confidence intervals of column 2 in Table 3.

**Fig 3:**
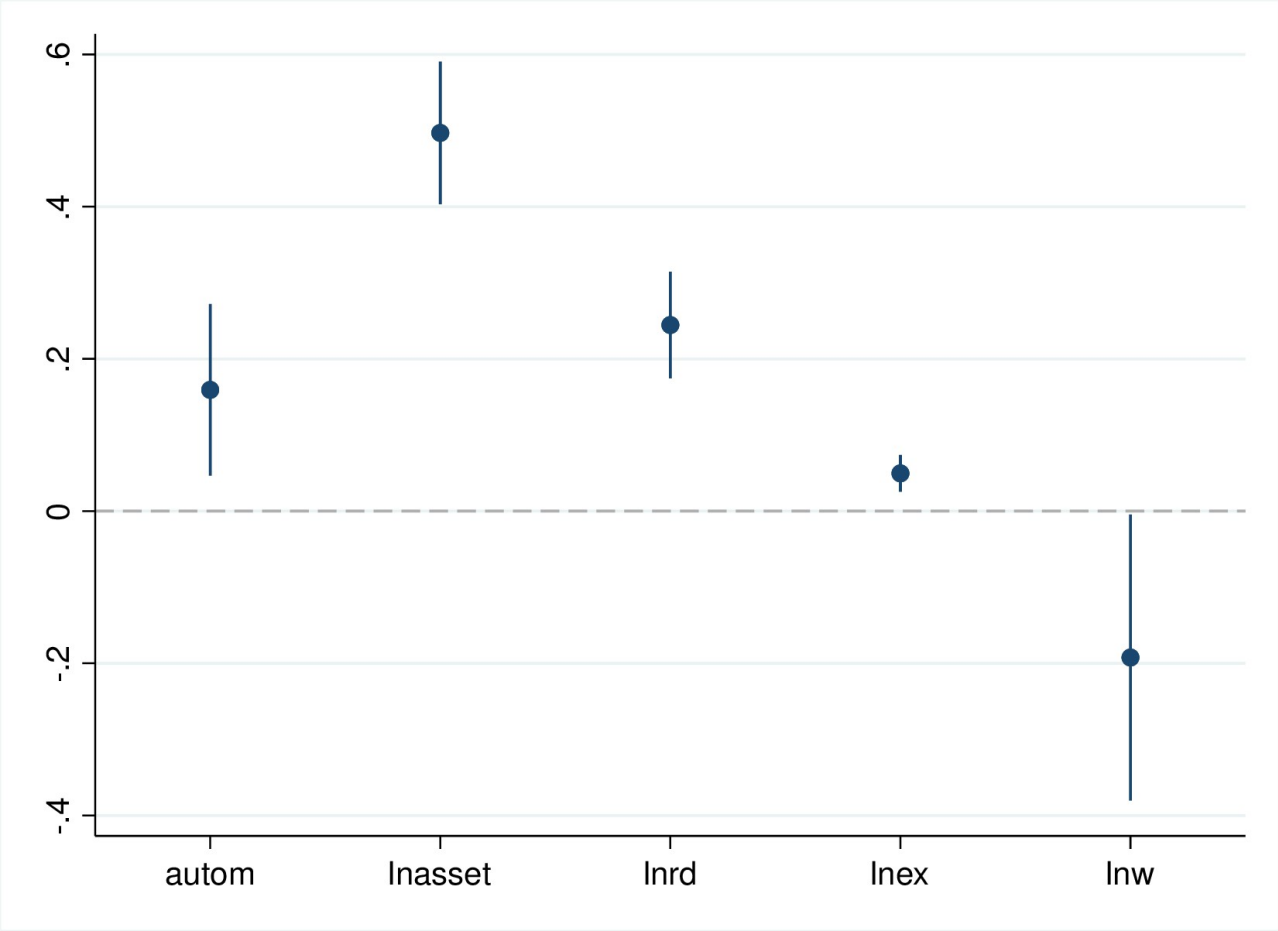
Estimated coefficients and confidence intervals of column 3 in Table 3.

The results of the baseline regression of the impact of automated machines in manufacturing firms on employment are shown in Table 3. Figs 1–3 show the regression coefficients and the confidence intervals of baseline regression. *autom* coefficient in column (1) is negative at the 1% statistical level, indicating that automated machine in manufacturing firms significantly reduce total employment. Columns (2)-(3) show that automated machine in manufacturing firms have a significantly negative and positive effect on employment of the low- and high-skilled labor force, respectively. Therefore, hypothesis 1 is verified.

**Table 3:** Results of baseline regression.

Variables	(1)	(2)	(3)
	ln*l*	ln*L*	ln*Z*
*autom*	-0.1690***	-0.2950***	0.1592***
	(-2.6203)	(-4.7015)	(2.7720)
ln*asset*	0.4962***	0.4831***	0.4969***
	(11.0706)	(10.1837)	(10.4232)
ln*rd*	0.1882***	0.1764***	0.2445***
	(5.8163)	(5.2078)	(6.8662)
ln*ex*	0.0616***	0.0681***	0.0496***
	(5.0739)	(5.3203)	(4.0314)
ln*w*	-0.3395***	-0.3787***	-0.1924**
	(-4.8236)	(-5.4963)	(-2.0119)
N	2677	2677	2677
adj. R^2^	0.9636	0.9590	0.9346
Firm FE	YES	YES	YES
Year FE	YES	YES	YES

*Notes*: *t* statistics in parentheses

* *p* < 0.1,

** *p* < 0.05,

*** *p* < 0.01

#### 4.2.2 Mechanism test

By referring to Baron and Kenny’s (1986) [31] approach to test the influencing mechanism of automated machines in manufacturing on the labor market based on the literature review and research hypotheses, this paper constructs the following mediating effect test model:

$$Me_{it}=\alpha +\beta autom_{it}+\theta X+v_{i}+\varphi_{t}+\varepsilon_{it}$$ (2)

$$\text{ln}l_{it}=\alpha +\beta autom_{it}+\theta X+\gamma Med_{it}+v_{i}+\varphi_{t}+\varepsilon_{it}$$ (3)

In this model, *Med*_*it*_ is the mediating variable, the control variable *X* is consistent with the baseline model, which also controls for individual and time fixed effects, *ε*_*it*_ is the residual term.

We first test the mechanism of output scale effect by using the logarithmic value of firm’s operating income ln*Y*_*it*_ as the mediating variable *Me*_*it*_. The results according to column (1) of Table 4, the effect of automated machine on output scale in manufacturing firms is non-linear, showing a U-shaped curve, and the extreme point is 0.9935. Column (2) shows that the effect of firm output scale on employment is significantly positive at the 1% statistical level, and the absolute value of the coefficient of the core explanatory variable(*autom*_*it*_)becomes smaller after adding the mediating variable (ln*Y*_*it*_) comparing to the baseline regression model in column (2), indicating that output scale plays a partial mediating effect in the effect of automated machines on total employment.

**Table 4:** Result of mechanism test.

Variables	(1)	(2)	(3)	(4)
	ln*Y*	ln*l*	*pro*	ln*l*
*autom*	-0.9661***	-0.1500**	0.1195**	-0.1071**
	(-3.5849)	(-2.5527)	(1.9824)	(-2.3077)
*autom*2	0.4862***			
	(3.4003)			
ln*asset*		0.2634***	0.1087**	0.5526***
		(6.2565)	(2.1871)	(15.0513)
ln*rd*	0.3545***	0.1215***	-0.0149	0.1805***
	(8.3950)	(3.3001)	(-0.3363)	(7.0927)
ln*ex*	0.1074***	0.0354***	0.0064	0.0650***
	(7.8680)	(3.1458)	(0.5238)	(6.3108)
ln*w*	0.1477***	-0.3985***	0.4927***	-0.0842*
	(3.6387)	(-6.3212)	(8.7917)	(-1.7333)
ln*Y*		0.3848***		
		(8.5540)		
*pro*				-0.5183***
				(-12.3007)
N	2677	2677	2677	2677
adj. R^2^	0.9616	0.9687	0.8766	0.9755
Firm FE	YES	YES	YES	YES
Year FE	YES	YES	YES	YES

*Notes*: *t* statistics in parentheses

* *p* < 0.1,

** *p* < 0.05,

*** *p* < 0.01

Secondly, we test the productivity effect. Taking labor productivity (*pro*_*it*_ = *Y*/*l*) as the mediating variable (*Me*_*it*_). The results are shown in columns (3) and (4) of Table 4. According to column (3) of Table 4, the coefficient of *autom* is significantly positive, indicating that automated machines in manufacturing enterprises will significantly improve the labor productivity of enterprises. We add the mediating variable *pro*_*it*_ based on the baseline regression in column (4) of Table 4. While the coefficients of *pro*_*it*_ and *autom* are both significantly negative in column (4), and the absolute value of the coefficient of automated machines in manufacturing enterprises become smaller, which indicates that productivity plays a partial mediating effect. Therefore, Table 4 shows that the productivity effect and output scale effect are the influencing mechanisms of the impact of automated machine on employment, which verifies hypothesis H_3_.

#### 4.2.3 Industry linkage effect test

Considering that there is a certain correlation effect between upstream and downstream industries in manufacturing, in order to more comprehensively examine the impact of manufacturing automated machines on employment, this paper examines its impact on the labor demand of upstream and downstream enterprises. Drawing on Zhu et al. (2020) [37], this paper measures the technological level of upstream and downstream manufacturing enterprises’ automated machines, denoted as *forward_autom*_*it*_, *backward_autom*_*it*_, respectively, which are constructed as follows:

$$forward\_autom_{it}=\Sigma_{j\neq s}(input_{jst}/\Sigma_{s}input_{jst})\times autom_{ijt}$$ (4)

$$backward\_autom_{it}=\Sigma_{j\neq x}(output_{jxt}/\Sigma_{s}output_{jxt})\times autom_{ijt}$$ (5)

Where *input*_*jst*_ means the intermediate goods obtained by industry *j* from upstream industry *s*, sum up all intermediate inputs obtained by industry *j* from upstream industry *s*, denoted as ∑_*s*_ *input*_*jst*_; *output*_*jxt*_ means the intermediate goods sold by industry *j* to downstream industry *x*, sum up all intermediate goods sold by industry *j* to all downstream industries *x*, denoted as ∑_*x*_ *output*_*jst*_. The data of *input*_*jst*_ and *output*_*jst*_ are obtained from the Input-Output Tables of 2012, 2017 and 2018, and the Input-Output Tables of these three years are used to calculate the direct consumption coefficients and direct distribution coefficients from 2012 to 2019. According to Zhu et al. (2020) [37], the direct consumption coefficients and direct allocation coefficients for 2012–2014, 2015–2017, and 2018–2019 were replaced with those for 2012, 2017, and 2018, respectively.

Based on the baseline model (1), *forward_autom*_*it*_ and *backward*_*autom*_*it*_ are regressed as the core explanatory variables, and the results are shown in Table 5. The results in columns (1) and (3) of Table 5 show that the coefficients of *forward*_*autom*_*it*_ and *backward*_*autom*_*it*_ are significantly positive at the 1% statistical level, and the coefficients of *forward*_*autom* and *backward*_*autom* in columns (2) and (4) of Table 5 are significantly negative, implying that the improvement of technological level of automated machines in upstream (downstream) enterprises has a negative effect on ln*l* of downstream (upstream) enterprises, which verifies hypothesis H_2_.

**Table 5:** Results of industry linkage effects.

Variables	(1)	(2)	(3)	(4)
	*autom*	ln*l*	*autom*	ln*l*
*forward*_*autom*	2.2039***	-0.4059**		
	(18.4176)	(-2.0079)		
*backward*_*autom*			0.4650***	-0.1386*
			(5.4963)	(-1.7044)
ln*asset*	-0.0108	0.4976***	-0.0193	0.4971***
	(-1.3751)	(11.0749)	(-1.5628)	(11.2441)
ln*rd*	-0.0022	0.1882***	-0.0230***	0.1918***
	(-0.6596)	(5.8211)	(-3.4615)	(5.9309)
ln*ex*	-0.0060***	0.0626***	-0.0114***	0.0638***
	(-3.5083)	(5.1744)	(-3.5120)	(5.2430)
ln*w*	0.0244***	-0.3434***	0.0303	-0.3431***
	(2.7796)	(-4.9052)	(1.6236)	(-4.8724)
N	2677	2677	2677	2677
adj. R^2^	0.8842	0.9635	0.6885	0.9634
Firm FE	YES	YES	YES	YES
Year FE	YES	YES	YES	YES

*Notes*: *t* statistics in parentheses

* *p* < 0.1,

** *p* < 0.05,

*** *p* < 0.01

### 4.3 Endogeneity control

The following endogeneity tests were done in this paper:

(1) Omitted variable problem. To address the endogeneity problem caused by omitted variables, this paper controls for interaction terms of firm and year, interaction terms of firm and industry, and interaction terms of year and industry based on the baseline model [33]. The regression results are reported in column (1) of Table 6, indicating that the effect of *autom* on *lnl* is significantly negative after controlling for the fixed-effects interaction term, and the coefficient of *autom* does not change significantly compared to the baseline regression results, implying that the endogeneity problem due to omitted variables is small.
(2) Reverse causality problem. Considering that there may be endogeneity between manufacturing enterprises’ automated machines and labor demand due to reverse causality, i.e., enterprises are prompted to invest in automated machines by either insufficient labor or escalating labor costs, and this paper constructs two instrumental variables to address this endogeneity problem. We construct the arithmetic mean (*hautom*) of the technological level of automated machines at the two-code industry level [36], and the regression results are shown in column (2) of Table 6. This instrumental variable passes the unidentifiable test, and there is no weak instrumental variable problem, i.e., the technological level of automated machines at the industry level is correlated with *autom* at the firm level, but not directly related to ln*l* at the firm level, consistent with the principle of setting instrumental variables. The results in column (2) show a negatively significant effect of *autom* on employment, which is consistent with the results of the benchmark regression.

**Table 6:** Endogeneity test.

Variables	(1)	(2)	(3)
	ln*l*	ln*l*	ln*l*
*autom*	-0.1699***	-0.5076**	-0.7106***
	(-2.6534)	(-2.0136)	(-4.5646)
ln*asset*	0.4973***	0.6267***	0.4988***
	(11.1182)	(30.9915)	(20.3887)
ln*rd*	0.1882***	0.0802***	0.2351***
	(5.8106)	(8.2877)	(10.9272)
ln*ex*	0.0615***	0.0904***	0.0818***
	(5.1110)	(13.4806)	(11.3036)
ln*w*	-0.3362***	-0.4762***	-0.5543***
	(-4.8638)	(-12.5788)	(-14.4892)
*id*_*year*	YES		
*code*_*year*	YES		
*id*_*code*	YES		
adj. R^2^	0.9636	0.7481	0.7599

*Notes*: *t* statistics in parentheses

* *p* < 0.1,

** *p* < 0.05,

*** *p* < 0.01

In this paper, referring to Goldsmith-Pinkham et al. (2020) [32] and Zhao et al. (2021) [33], we use the share shift method to construct the Bartik instrumental variable *autom*_*iv*_*ijt*_, expressed as Eq (6):

$$autom\_iv_{ijt}=\Sigma_{i\epsilon J}autom_{ijt_{0}}\times \left(1+g_{it}\right)$$ (6)

We denote the initial year by *t*_0_, i.e., 2012 in this paper. For any firm *iϵJ*, $autom_{ijt_{0}}$ denotes the technological level of automated machine of firm *i* corresponding to industry *j* in the initial year *t*_0_. *g*_*it*_ denote the growth rate of technological level of automated machine of industry *j* in year *t* relative to the initial year *t*_0_. Based on data availability, we use the growth rate of fixed assets of the two-bit code industry relative to the initial year to characterize *g*_*it*_. This instrumental variable is obtained simply by computing the initial state $autom_{ijt_{0}}$ and the exogenous growth rate *g*_*it*_, which will be highly related to *autom*_*it*_. This Bartik instrumental variable is not correlated with other residual terms affecting firms’ *autom* and meets the basic requirements of instrumental variables. Column (3) of Table 6 reports the results of the two-stage least squares estimation. The coefficient of the core explanatory variable is significantly negative at the 1% statistical level and the absolute value of the coefficient of *autom* is significantly higher compared to the result of benchmark regression, which implies the existence of a reverse causal effect and the results of the benchmark regression in this paper remain reliable.

### 4.4 Robustness test

To enhance the robustness of the results of this paper, the following tests were done:

(1) Placebo test. In addition to endogenous issues, there may be another challenge in identifying the relationship between automated machines and the labor. Due to factors such as national employment policy orientation and population aging trend, there may be a trend of rising labor cost in manufacturing enterprises, and with the rapid development of new technologies such as information technology and digital technology, the labor demand of manufacturing enterprises shows a decreasing trend. In this scenario, the above estimates may confuse the employment substitution effect triggered by the automated machines in manufacturing with the declining trend of employment in manufacturing firms. To deal with these concerns, this paper examines whether labor force employment in manufacturing firms before 2012 is related to the future level in automated machines through a placebo test. Using *autom*_*it*_ from 2012 to 2019 to regress the number of jobs in manufacturing from 2004 to 2011 respectively, it is theoretically unlikely that future *autom* will affect the past employment, and if significant regression results are not obtained, the trend correlation between *autom*_*it*_ and employment in manufacturing can be ruled out. Column (1) of Table 7 reports the estimation results and it can be seen that the estimated coefficients of the core explanatory variable are not significant, indicating that *autom* is not related to ln*l*_*before*, and there is no trend correlation between the two, ensuring the robustness of the results again.
(2) Drawing on Yu et al. (2020) [38], the 2.5% of maximum and minimum values of the explanatory variable ln*l* are excluded, and the results are shown in column (2) of Table 7, where the coefficient of *autom*_*it*_ is negative and significant statistically, indicating that the results of the baseline regression are robust. (3) We use two-stage least squares regression taking lagged one period of *autom* (*L*.*autom*) as the instrumental variable [34]. Column (3) of Table 7 demonstrates the results, and the coefficient of *autom* is significantly negative, which is consistent with the baseline regression results.

**Table 7:** Robustness tests.

Variables	(1)	(2)	(3)
	ln*l_before*	ln*l*	ln*l*
*autom*	-0.1545	-0.1605**	-0.4744***
	(-1.5374)	(-2.5259)	(-7.0233)
ln*asset*	-0.1571*	0.4863***	0.5230***
	(-1.8857)	(10.8856)	(24.5150)
ln*rd*	-0.0083	0.1961***	0.2282***
	(-0.1878)	(6.2443)	(11.4670)
ln*ex*	0.0559***	0.0637***	0.0813***
	(2.6408)	(5.2777)	(13.1283)
ln*w*	0.2572**	-0.3336***	-0.6170***
	(2.3405)	(-4.6668)	(-18.7281)
N	7.1346***	-3.0074***	-1.2602***
adj. R^2^	(4.2947)	(-2.7571)	(-2.9487)
Firm FE	YES	YES	YES
Year FE	YES	YES	YES

*Notes*: *t* statistics in parentheses

* *p* < 0.1,

** *p* < 0.05,

*** *p* < 0.01

### 4.5 Further testing

Changes in total employment do not yet accurately capture the structural changes involved, and because automated machine has heterogeneous effects on different types of tasks [1], this paper further decomposes the total employment into different jobs. Since the annual reports of listed manufacturing companies disclose the number of employees in different positions, including production personnel (ln*l*_*prod*), financial personnel (ln*l*_*finan*), R&D technicians (ln*l*_*tech*), sales personnel (ln*l*_*sale*), and administrative personnel (ln*l*_*admin*). The administrative positions provide support to the business departments, referring to the labor force other than senior executives, production personnel, technical and R&D personnel, and financial personnel. This paper examines the impact of automated machines in manufacturing on the employment of five positions, and the regression results are shown in Table 8. The effects of *autom* on the employment of production personnel, finance personnel, and administrative personnel are significantly negative, and the negative effect on production personnel is the largest and most significant; the effects of *autom* on the employment of R&D technicians are significantly positive, and the effect of *autom* on sales personnel is not significant. The results mean that automated machines in manufacturing will significantly increase the demand for highly skilled labor such as R&D technicians, which is highly complementary to automated machines. And the result is consistent with to the capital-skills complementarity hypothesis, and with the innovation of automation technology and the improvement of intelligence, there will be a greater demand for highly skilled labor that can complement automated machines, such as high-skilled labor in the fields of automation and intelligent research and design, equipment manufacturing and equipment application [24, 25]. Production personnel have low complexity and repeatability of work content, which can be easily replaced by automated machines. Because the rapid spread of intelligent financial systems and office management systems in enterprises in recent years has greatly improved the efficiency of financial personnel and administrative personnel, the demand for these two types of labor is reduced [39]. The work tasks of sales personnel are mainly to communicate with customers, discover their needs, recommend products that can meet their needs, and maintain long-term relationships with customers. This type of work task is difficult to be replaced by machines, so automated machines have no significant effect on the employment of sales personnel.

**Table 8:** Further research.

Variables	(1)	(2)	(3)	(4)	(5)
	ln*l_prod*	ln*l_finan*	ln*l_tech*	ln*l_sale*	ln*l_admin*
*autom*	-0.3222***	-0.1209**	0.1516***	-0.0447	-0.1057*
	(-4.8322)	(-2.2296)	(2.6761)	(-0.5704)	(-1.7659)
ln*asset*	0.4974***	0.4570***	0.4986***	0.4504***	0.3707***
	(10.2481)	(10.0738)	(10.2211)	(7.0251)	(5.6409)
ln*rd*	0.2259***	0.1040***	0.2547***	0.1645***	0.1895***
	(6.3641)	(3.2617)	(6.5786)	(3.7789)	(4.4342)
ln*ex*	0.0734***	0.0485***	0.0489***	0.0415***	0.0435**
	(4.6848)	(3.2163)	(3.9742)	(2.7020)	(2.2583)
ln*w*	-0.4029***	-0.0607	-0.1830*	-0.1855**	-0.2093***
	(-4.6124)	(-0.7219)	(-1.9080)	(-2.1174)	(-3.0158)
N	2664	2520	2677	2641	2636
adj. R^2^	0.9524	0.9385	0.9343	0.9392	0.8762
Firm FE	YES	YES	YES	YES	YES
Year FE	YES	YES	YES	YES	YES

*Notes*: *t* statistics in parentheses

* *p* < 0.1,

** *p* < 0.05,

*** *p* < 0.01

## 5. Discussion

This paper uses data from Chinese manufacturing listed companies from 2012 to 2019, based on the task-based model as well as industry linkage effect and mediation effect, to study the impact of automated machines on employment and the mechanism.

First, automated machines in manufacturing firms significantly reduce total employment. Column (1) of Table 3 shows that the effect of *autom* on *lnl* is significantly negative with a coefficient of -0.1690, indicating that manufacturing firms have a substitution effect on total employment, which is consistent with the findings of Wang (2014) [40, 41], and Acemoglu and Restrepo (2018) [1]. This is due to the fact that firms invest in more automated machines, generating the effect of machine substitution on labor and thus reducing the demand for labor.

The change in total employment does not yet accurately capture the structural changes, and a structural decomposition of total employment is required. Columns (2) and (3) of Table 3 show the effect of *autom* on the structure of employment. The coefficients of ln*L*, ln*Z* are -0.2950 and 0.1592 respectively, and both are significant at the 1% statistical level. The results indicate that automated machines in manufacturing firms have a destructive effect on low-skilled labor and an employment creating effect on high-skilled labor. And the destructive effect on low-skilled labor is outweighs the creative effect on high-skilled labor. This also implies that in the sample studied in this paper, the redundancy of low-skilled labor is relatively serious, there is more room for machine substitution, and the overall employment structure is shifting to high quality with improving the technological level of automated machines and increasing the demand for high-skilled labor. Thus, while increased input of automated machines can have a negative impact on total employment, the creation effect on a highly skilled workforce can mitigate the negative impact.

Second, the output scale effect and productivity effect are the influencing mechanisms of the automated machine on employment. Column (1) of Table 4 shows that the automated machines of manufacturing firms will reduce output scale if *autom* is smaller than 0.9935 and will increase the output scale if *autom* is bigger than 0.9935, because automated machine input for enterprises in the short-term requires a large amount of money, with significant risks and a long time to obtain returns. The cost effect of enterprise automated machine input results in a decrease in output scale. As the improvement of technological level of automated machine, the production efficiency brought by automated machine can increase the enterprise output scale, stimulating enterprises to add capital and labor to expand production scale. This is consistent with the research results of Graetz and Michaels (2018) [28]. Column (2) of Table 4 shows that the effect of the output scale on employment is significantly positive, implying that the expansion of firm output scale requires matching more labor, and therefore, automated machines of manufacturing firms will have a creation effect on employment by increasing firm output scale.

Column (3) of Table 4 shows that automated machines of manufacturing firms significantly improve labor productivity. This is due to the fact that automated machines improve the overall productivity of a company’s workforce by replacing low-level, repeatable, and simple tasks on the one hand. And on the other hand, the effective matching and collaboration between a highly skilled workforce and automated machines can enhance the productivity of a highly skilled workforce. The productivity effect (*pro*_*it*_) on employment in column (4) is significantly negative at the 1% statistical level, which may be due to the increased productivity of the firm’s labor force, which will reduce the demand for labor in order to save labor cost. Thus, it can be concluded that the automated machines in manufacturing enterprises have a substitution effect on labor demand by enhancing the productivity of enterprises. In summary, the combination of the output scale expansion effect and productivity enhancement effect leads to the substitution effect of automated machines on employment.

Third, the result implies that automated machines in upstream (downstream) manufacturing firms will have a substitution effect on the total labor force in downstream (upstream) firms. Columns (1) and (3) of Table 5 imply that the increase of *autom* in upstream (downstream) enterprises of manufacturing industry will promote the technological level of downstream (upstream) enterprises in automated machines, and the coefficient of *forward*_*autom*_*it*_ is larger than the coefficient of *backward*_*autom*_*it*_, indicating that the more upstream enterprises in manufacturing invest in automated machines, the greater the labor productivity effect on downstream enterprises, which may be because products of upstream enterprises are more widely used and more malleable, while downstream enterprises have more intermediate inputs, are more influenced by upstream enterprises, and need to match the technological progress of upstream enterprises more. In columns (2) and (4) of Table 5, the coefficients of *forward*_*autom*_*it*_ and *backward*_*autom*_*it*_ are negatively significant, and the coefficient of *forward*_*autom*_*it*_ (-0.4059) is bigger than that of *backward*_*autom*_*it*_ (-0.1386), which shows that automated machines in upstream manufacturing enterprises has a greater substitution effect on the labor demand of downstream enterprises by affecting the *autom*. The automated machines in either upstream or downstream enterprises in manufacturing will enhance the productivity and automated level of other industries in manufacturing through the technology spillover effect, which will have a substitution effect on the labor force in other industries in manufacturing.

## 6. Conclusions

This paper uses micro data from listed manufacturing companies and draws on the idea of the task-based model to theoretically analyze and empirically test the impact and mechanisms of automated machines on the employment, and the study concludes that: (1) The automated machines in manufacturing enterprises has a substitution effect on the total labor force and has a substitution effect on low-skilled labor and a creation effect on high-skilled labor. (2) Further analysis shows that automated machines in manufacturing firms have a creation effect mainly on R&D and technical staff, a non-significant effect on sales staff, and a destructive effect on production, administrative and financial staff. (3) The results of the mechanism test indicate that the main mechanisms of the employment effects of automated machines in manufacturing firms are productivity effects and output scale effects. (4) About the industry linkage effect test, we found that the automated machines in upstream (downstream) enterprises of the manufacturing industry has a negative effect on the labor demand of downstream (upstream) enterprises by improving the technological level of automated machines in downstream (upstream) enterprises, and the correlation effect of automated machines in upstream enterprises on the downstream labor is greater than that of the opposite situation.

Based on this, the policy implications of this paper are as follows:

(1) The government should encourage and support the input of automated machines, pay attention to the transmission and synergy effect of the industrial chain, and vigorously promote the technological level of automated machines in manufacturing. This paper finds that automated machines can increase the number of R&D and technical personnel and promote employment quality. At the same time, manufacturing automated machines can improve the labor productivity and increase the output level of manufacturing firms in the long term. Therefore, the government can encourage and support manufacturing enterprises to invest in automated machines through preferential policies and initiatives such as taxation, low-interest loans, and innovation subsidies, and increase the input and output of basic research, applied research, and experimental development in the field of automation technology. Meanwhile, it is necessary to pay attention to the promotion effect of automated machines of the upstream and downstream enterprises in the manufacturing industry on technological progress in one industry, to strengthen the technical exchange and cooperation between the upstream and downstream enterprises in the manufacturing industry, and to promote the expansion of the input scale in automated machines in the manufacturing industry by making use of the synergistic effect of the industrial chain and technological spillover effect of the upstream and downstream industries.
(2) There is an urgent need to upgrade the skill level of the workforce and vigorously cultivate a highly skilled workforce to cope with the impact of automated machines on the labor market. The research in this paper shows that automated machines on the one hand will cause shocks to manufacturing employment, however, such shocks are mainly for production, finance, and administrative personnel, who perform relatively simple and repetitive tasks that are easily replaced by the automated machines. The government should improve the social insurance system, adopt active employment assistance for the unemployed, pay attention to the improvement of the skill level of low-skilled labor, and induce them to transform into high-skilled labor matching this technological progress through skill subsidies, job training, and re-education, or engage in new occupations spawned by automated machines (e.g. robot designers, data labelers, intelligent robot trainers, intelligent equipment repairers, human-machine cooperation dispatchers, etc.), while creating opportunities to promote their transfer to industries or services that combine traditional industries with new industries. On the other hand, increased investment in automated machines will increase the demand for highly skilled labor such as research technicians. At present, there is a shortage of talents in the field of automation technology in China, and the shortage of top talents is even greater. It is necessary to adopt the policy of combining attraction and training, using salary incentives, optimizing urban resources support, expanding industrial development space, and other ways to attract top talents at home and abroad to carry out scientific research and technology development in the field of automation and intelligent technology, and it is also necessary to increase the high-skilled labor force through school curriculum education and enterprise skills training, actively promote school-enterprise cooperation, and enhance the conversion rate of the achievements of researchers.
(3) We should excavate the active role of the productivity effect and output scale effect in automated machines. The positive effects of productivity and output scale on the labor market should be given full play. The government should give certain financial and tax benefits in the expansion of output scale and productivity enhancement to alleviate the financing constraints and cost pressure of enterprises, strengthen the effective collaboration between automated machines and labor, drive the positive effects of productivity and output scale effect on employment, and promote high-quality employment in the manufacturing industry.

The research in this paper provides a reference for addressing the trend of machines substituting humans, promoting high-quality employment, and balancing the development of new technologies and new business with the protection of employment and people’s livelihood. This paper can also be extended in the following aspects. First, to study the impact of automated machines on income inequality in the manufacturing industry. Second, to study the impact of automated machines in the service industry on the labor market, and to explore the correlation effects of automated machines in the manufacturing industry and the service industry on employment and wages.
